# Annotations

**Published:** 1896-10-03

**Authors:** 


### Annotations.

**Anti-Vaccination: A New Departure.**

It is stated that the "Coventry anti-vaccinators contemplate establishing a medical service of their own, in order that they may be independent of doctors who uphold vaccination." The Coventry anti-vaccinators will, of course, do as they please. They may, however, be reminded of the undoubted fact that practically every doctor of capacity and experience throughout the country is a convinced vaccinator; and they will, therefore, have their choice of doctors narrowed down to two strictly limited classes, namely, those doctors who, for the sake of gain, will stifle their honest convictions, and those who have no convictions to stifle, but instead thereof are the weak victims of exploded "fads." Even anti-vaccinators, one would suppose, must know that there are "doctors and doctors," and they cannot but see that to employ doctors of all-round incompetence and defective character merely because they are willing to pronounce the shibboleth of "anti-vaccination" is a very high price to pay for the indulgence of what a better intelligence has proved to be a mere whim. There is an aspect of the question which is important to the public and the medical profession. If Coventry sets the example, other towns will, no doubt, follow in its wake; and organisations of the kind indicated may bring about in many towns conditions similar to those which so recently and disastrously prevailed at Gloucester. It is eminently desirable to prevent such serious results. Apparently they will not be prevented by the resort to sterner compulsion, and other methods must therefore be sought. It is for local governments to take upon themselves the necessary duty of enlightening the public mind on this seriously important question. Instruction is what is needed; competent, systematic, persevering instruction. For this purpose lecturers, of a class similar to University Extension lecturers, might be usefully employed. There are scores of clever, competent young medical men who might profitably, and no doubt willingly, spend a year or two in work of this kind. We commend the suggestion to local authorities.

**Ladies and Bagpipes.**

"A ladies' society," we are informed, "has been started for the study of the bagpipes." It is not stated whether the ladies are to "study" the pipes as expounded by men pipers, but presumably "study" here means practice, and the ladies of the society, no doubt, intend to "play" the pipes themselves. The physiology of ten years ago would have uttered notes of warning of a very portentous character. Pictures of puffed-out cheeks, starting eyes, emphysematous lungs, and depressed diaphragms would have been conjured up before excited visions; and serious attempts would have been made to put a peremptory stop to the whole business. But physiology in these later times has become wiser. It has learnt, as mere common sense learnt long ago, that when the ladies have made up their minds to try any given experiment, that experiment will be tried, even though all the swords of all the sciences in civilisation should be brandished before their eyes. If, then, there really has been formed a "ladies' society for the study of the bagpipes," we of the present day make no objection, but simply make up our minds to wait, and see what the results will be. That the female piper will ever equal the male in the energy of her performances we do not expect, though it is by no means improbable that she will equal or even surpass him in the hideousness of the "music" she will produce.

**Shyness!**

To be shy is a terrible drawback, and has in many cases entirely prevented men from occupying positions in life the duties of which they could otherwise have perfectly well performed. Indeed, as was well said by Dr. Harry Campbell in discoursing on the subject at Carlisle, when a paroxysm of shyness causes such symptoms as faintness, nausea, twitchings, and aphasia; when it compels the sufferer to shun society—to stand aside from the battle of life and to degenerate into a suspicious self-centred hypochondriac—then we must allow that shyness is a disease deserving of the most careful study by the physician. Shyness is a species of fear, an unreasoning dread not altogether unlike the curious dread of crossing open spaces by which some people are affected, and, like other mental twists and peculiarities, is apt to run in families. Shyness is not, however, in any way the result of a sense of inferiority. "That the shy man is not necessarily distrustful of his powers is shown by the fact that he may become aggressive—even offensively so—in his writings. Some of our most self-assertive and combative writers have been shy men." Shyness may even arise from pride, as when the proud and self-conscious schoolchild is shy at being made to display its ignorance in class. We are probably far too apt to look upon shyness as a little feminine weakness. Perhaps women hide their feelings better, but men usually appear to suffer more acutely from this malady, if one may so call it, than women—and especially married women—do. As Jerome K. Jerome says, "A woman will enter a concert-room late, interrupt the performance, and disturb the whole audience without moving a hair, while her husband follows her, a crushed heap of agonising misery." In regard to the causes of shyness, it is curious how potent an influence in its production is the fact of being looked at. Walking up the aisle of a church during service, or across a large room where people are sitting around, or being singled out for conversation at a dinner party amid expectant silence, is a terrible ordeal to the morbidly shy person; and if the sufferer is, in addition, cursed with a tendency to blush, his miseries are increased tenfold. It is worth remembering, that shyness may not always lead to silence. Just as it may lead to certain tricks of manner, to giggling and nervous laugh, or to stupid *gaucherie*, so it may lead to excessive talking: a girl may drown her dread by constant chatter. Lastly, shyness is no small affair to be laughed at or made light of, for the dislike so engendered of mixing freely with his fellows handicaps the sufferer in the race of life; it cuts him off from active commerce with the world, so that the shy man is apt to develop into a hypochondriac—to become absorbed in the study of the functions of his own body—and drifts into the morbid and eccentric recluse. We have said that shyness is a species of fear. Dr. Campbell says the essential factor is "the soul shuddering to feel itself naked"—a picturesque though perhaps not a very convincing way of putting it.

